# A Narrative Review of Perioperative Peripheral Nerve Injuries After Major Surgery: Clinical Recognition, Electrodiagnostic Evaluation, and Rehabilitation Implications

**DOI:** 10.7759/cureus.103887

**Published:** 2026-02-19

**Authors:** Jaime Vallejos G, Bruno Fajardo C

**Affiliations:** 1 Physical Medicine and Rehabilitation, Hospital Clínico de la Universidad de Chile, Santiago, CHL

**Keywords:** electrodiagnosis, electromyography, iatrogenic injury, nerve conduction studies, patient positioning, perioperative nerve injury, peripheral neuropathy, rehabilitation

## Abstract

Perioperative peripheral nerve injuries (PPNIs) are complications of surgical and procedural care and may cause neuropathic pain and motor or sensory deficits that delay functional recovery. This narrative review summarizes common patterns of PPNI involving the extremities, practical bedside recognition, the role and timing of electrodiagnostic testing, and key rehabilitation considerations. Upper-limb involvement most often affects the brachial plexus, ulnar nerve, and median nerve. In contrast, lower-limb presentations commonly involve the lateral femoral cutaneous, sciatic, and common fibular nerves, often in the setting of prolonged procedures and positioning. Electrodiagnostic studies complement the clinical examination by refining localization, distinguishing conduction block from axonal injury, and informing prognosis and follow-up planning. From a rehabilitation perspective, early detection supports prompt mitigation of ongoing compression or traction, symptom management, protection of insensate tissues, and timely functional strategies (including orthoses and task-oriented therapy) while recovery is monitored over time.

## Introduction and background

Perioperative peripheral nerve injuries (PPNIs) are clinically important complications of major surgery and invasive procedures [1]. When they occur, they may present with neuropathic pain, sensory loss, weakness, or a combination of these features that can significantly delay mobilization, prolong hospitalization, and compromise return to function [2]. Although many cases improve over time, a subset evolves into persistent impairment, with substantial consequences for activities of daily living, work participation, and quality-of-life outcomes that are highly relevant to rehabilitation teams [3-6].

PPNIs can be easily missed in the first postoperative hours or days. Sedation, residual anesthetic effects, delirium, and analgesics may blunt symptom reporting and reduce the reliability of the neurologic examination [7]. Early complaints may also be attributed to expected postoperative symptoms, such as pain, heaviness, tingling, or short-lived numbness after regional anesthesia, and thus may be dismissed as warning signs. Even when a focal deficit is recognized, clinicians may initially struggle to localize the problem and determine whether it reflects a peripheral mononeuropathy/plexopathy, a central event (stroke or spinal cord pathology), or a structural complication such as hematoma or compartment syndrome, each with different time sensitivity and management priorities [8]. In selected high-risk settings, intraoperative neurophysiological monitoring may provide an earlier signal of position-related nerve compromise, before clear clinical deficits emerge [7,8].

The mechanisms behind PPNIs are usually not attributable to a single cause. Patient positioning plays a major role: nerves may be compressed against rigid surfaces, stretched across joints, or exposed to prolonged focal pressure, particularly during long procedures [9,10]. Iatrogenic injury can also occur more directly through surgical dissection or retraction, tourniquet-related ischemia, or needle/catheter placement in the context of regional anesthesia [11,12]. Systemic and patient-related factors often add a layer of susceptibility, including hypotension or hypoperfusion, hypothermia, metabolic disturbances, and conditions such as diabetes, thyroid disease, alcohol-related neuropathy, pre-existing (sometimes subclinical) entrapment neuropathies, or low body mass index with reduced soft-tissue cushioning [5]. Intraoperative physiologic monitoring, such as somatosensory evoked potentials, has been used to examine how hemodynamics and upper-extremity positioning may contribute to position-related neurapraxia, a transient conduction block caused by focal demyelination without axonal disruption, during prone spine surgery [13]. Taken together, these observations support a clinically plausible “double hit” model, conceptually related to the double-crush phenomenon, in which a previously vulnerable nerve becomes symptomatic when exposed to perioperative stressors [10]. Additionally, not all perioperative neuropathies are purely mechanical; a distinct and potentially treatable subset may reflect postsurgical inflammatory neuropathy [8].

Clinically, PPNIs tend to present in fairly consistent patterns [7]. In the upper extremity, the most frequently involved nerves are the ulnar nerve, the median nerve, and the brachial plexus, often linked to shoulder abduction/external rotation, arm-board positioning, and elbow flexion or direct pressure at the medial epicondyle [7,9]. In the lower extremity, meralgia paresthetica (lateral femoral cutaneous nerve), sciatic neuropathy, and common fibular (peroneal) neuropathy are frequently encountered, particularly with prolonged immobility, lithotomy positioning, Trendelenburg, or external compression near the fibular head [9,14]. Peripheral nervous system complications have also been reported after major cardiac surgery, highlighting how procedure type and perioperative exposures can shape risk [15]. Because these presentations can resemble radiculopathy or even central etiologies, careful bedside localization, integrating sensory distribution, peripheral nerve versus myotomal weakness, and reflex patterns, is the cornerstone for initial triage and localization [8].

Electrodiagnostic testing, such as nerve conduction studies and electromyography (NCS/EMG), has a key role in confirming lesion localization, clarifying the underlying process (conduction block/neurapraxia versus axonal loss), estimating severity, and informing prognosis [16]. In practice, early NCS/EMG can be falsely reassuring, whereas denervation becomes evident only after Wallerian degeneration. Interpretation should account for technical factors (e.g., segment length, temperature, stimulation sites) and for serial comparability when follow-up studies are planned [16,17]. Needle EMG findings, such as fibrillation potentials and their association with subsequent muscle atrophy, can further help gauge injury severity and track recovery [18]. When electrodiagnostic testing is scheduled appropriately and paired with careful serial bedside examinations, clinicians can avoid false reassurance from premature studies and better tailor early protection, pain management, and functional restoration strategies [16,17].

These challenges necessitate a review that links bedside recognition with electrodiagnostic timing and early rehabilitation decisions [16,17]. This narrative review synthesizes common perioperative patterns of peripheral nerve injury, emphasizes practical bedside-oriented localization, summarizes electrodiagnostic timing and interpretation, and proposes a stepwise clinical approach to reduce missed diagnoses and support timely, targeted rehabilitation.

Methods

A targeted, non-systematic literature search informed this narrative review. We searched PubMed/MEDLINE and Google Scholar from database inception through September 2025 using combinations of the following terms and keywords: PPNI, periprocedural neuropathy, iatrogenic nerve injury, postoperative neuropathy, patient positioning, lithotomy, Trendelenburg, regional anesthesia, nerve block complications, electrodiagnosis, nerve conduction studies, and electromyography. Because this was a narrative (non-systematic) review, we did not apply prespecified inclusion/exclusion criteria, provide reproducible database-specific search strings, or perform a formal risk-of-bias assessment for individual studies. We prioritized English-language publications and clinically oriented evidence, including narrative reviews, observational studies, case series, and relevant guidelines. Additional references were identified through manual screening of bibliographies and by tracking citations in key papers. The retrieved literature was synthesized narratively, with emphasis on common injury patterns, plausible mechanisms, practical bedside assessment, electrodiagnostic timing and interpretation, prevention strategies, and rehabilitation implications. This targeted approach was chosen to support a clinically oriented synthesis rather than a comprehensive systematic appraisal of all available evidence.

## Review

Clinical relevance and scope

Why PPNIs Matter for Function and Rehabilitation

PPNIs matter clinically for two main reasons: they are often preventable, and they can meaningfully derail functional recovery [7]. Even “minor” sensory changes may interfere with hand function, balance, and safe ambulation. In contrast, motor deficits can lead to obvious disability, such as reduced grip strength, foot drop, gait instability, or difficulty with transfers [7,17]. Neuropathic pain, which may be prominent even when weakness is modest, can further limit early participation in mobilization and therapy, disrupt sleep, and increase reliance on opioids [17,19]. For rehabilitation teams, delayed recognition can result in lost time to protect an at-risk nerve, avert secondary problems (e.g., contractures, falls, overuse injuries), and initiate practical interventions such as splinting/orthoses, graded activity progression, and task-specific retraining [7,17]. Taken together, these impacts highlight why early functional assessment is as important as neurologic localization in the perioperative setting [7,17].

Beyond the individual patient, PPNIs can contribute to longer hospitalizations, greater resource use, and more complex follow-up involving multiple specialties. A timely, structured assessment facilitates triage by distinguishing peripheral nerve injury from central nervous system causes or urgent surgical complications, and supports coordinated care among surgery, anesthesia, neurology, and physical medicine and rehabilitation. In day-to-day practice, rehabilitation clinicians are often well-positioned to detect subtle deficits during early mobilization, translate localization into functional goals, and guide symptom management as neurologic recovery progresses [7,19].

Definitions and Practical Terminology

In this review, PPNI refers to a new postoperative or periprocedural dysfunction affecting a peripheral nerve or plexus that arises in temporal association with surgery or another invasive intervention [11]. This includes injuries related to positioning, surgical handling (e.g., retraction, dissection, traction), tourniquet exposure, and complications associated with regional anesthesia, such as needle/catheter trauma or compressive hematoma [11]. We use the term "periprocedural" more broadly to encompass comparable injuries after nonoperative invasive procedures, such as prolonged endoscopy or interventional radiology, in which the mechanisms and diagnostic approaches often overlap [11]. These definitions are intended to ensure consistent terminology across surgical and non-surgical perioperative contexts.

For localization, we use conventional clinical categories: mononeuropathy for a single nerve, plexopathy for brachial or lumbosacral plexus involvement, and multifocal neuropathy when deficits span more than one peripheral nerve territory [10]. Because early postoperative symptoms can resemble radiculopathy or even central nervous system events, accurate localization depends on the pattern of motor deficits, sensory distribution, and reflex changes rather than symptom descriptors alone [10]. When discussing severity and prognosis, we frame injuries in practical terms, neurapraxia/conduction block versus axonal loss, since this distinction directly informs electrodiagnostic timing and the pace and priorities of rehabilitation [20,21].

Overview of Differential Diagnosis in the Early Postoperative Period

A practical early postoperative differential starts with a simple premise: new neurologic symptoms are not synonymous with peripheral nerve injury. When deficits are acute or evolving, especially if accompanied by severe pain, swelling, altered mental status, or sphincter dysfunction, clinicians must first consider central etiologies (such as stroke or spinal cord pathology), neuraxial complications (including epidural hematoma after neuraxial anesthesia or cauda equina syndrome), and limb-threatening problems like compartment syndrome or vascular compromise [22,23]. At the same time, apparently “peripheral” findings may still be iatrogenic in a more local sense, arising from external compression by dressings or casts, tight positioning devices, postoperative edema, or a hematoma within or near the operative field [22,24]. This triage-first approach helps prioritize diagnoses in which delay may be neurologically or surgically decisive.

After urgent conditions have been reasonably excluded, the bedside neurologic examination becomes the most useful tool for separating peripheral from non-peripheral causes [16]. A pattern that fits a single nerve or plexus, focal weakness in a peripheral distribution with sensory change that respects cutaneous territories, supports peripheral localization [16]. In contrast, cortical signs, bilateral or diffuse deficits, a clear sensory level, or upper motor neuron findings should shift the evaluation toward central etiologies [16]. One practical challenge is that transient sensory change after regional anesthesia can mask early PPNIs; for that reason, deficits that persist beyond the expected duration of block resolution, new motor involvement, or symptoms that worsen after an initially stable period should trigger prompt reassessment and, when timing allows, referral for electrodiagnostic evaluation [25,26]. This principle is revisited in the diagnostic pathway below to reduce missed or delayed recognition.

Pathophysiology and risk factors

Mechanisms of Injury

PPNIs typically reflect the combined effects of multiple factors rather than a single, isolated event [9,10]. Although these mechanisms frequently coexist clinically, they are described separately below for conceptual clarity. Compression is one of the most common contributors. It occurs when a nerve is exposed to sustained focal pressure against a rigid surface, such as the edge of an operating table, an arm board, or stirrups, or when it is pinched beneath tight padding, straps, or postoperative dressings [10]. Prolonged compression can compromise intraneural microcirculation, increase endoneurial pressure, and damage myelin, leading to conduction failure that may be reversible if the pressure is recognized and relieved early [9,10].

Stretch or traction becomes relevant when nerves are elongated beyond physiologic tolerance, particularly across mobile joints or at fixed anatomic tether points [9]. Positions that increase shoulder abduction with external rotation, cervical extension or rotation, or excessive hip flexion and abduction can place tension on the brachial plexus or lumbosacral structures [9]. Traction often coexists with compression, for example, when an extremity is restrained while also positioned to increase nerve tension [9]. Clinically, traction is often suspected after lengthy procedures, major intraoperative repositioning, or when deficits map to territories known to be vulnerable to stretch, such as the brachial plexus [9].

Ischemia and hypoperfusion can further lower the threshold for injury [10]. Systemic hypotension, anemia, hypovolemia, hypothermia, and prolonged operative time may reduce nerve perfusion and oxygen delivery [10]. In patients with limited physiologic reserve or pre-existing neuropathic vulnerability, even modest reductions in perfusion can convert an otherwise tolerable degree of compression or stretch into clinically apparent neuropathy [10]. Ischemic mechanisms are also relevant when a nerve is compressed within a confined compartment (intraneural ischemia) or when there is regional vascular compromise [10].

Direct trauma encompasses a different set of scenarios: injury during dissection, aggressive retraction, traction on tissue planes containing nerves, or inadvertent laceration [10,11]. It also includes iatrogenic injury related to instruments or procedural devices (e.g., cannulation, endoscopic positioning apparatus) and perioperative equipment such as tourniquets, which may produce a combined compressive and ischemic insult depending on pressure and duration [12,27]. Because direct trauma more often involves axonal disruption, it is typically associated with a longer recovery course. It should be considered when deficits align closely with the surgical field or a recognized high-risk maneuver [11]. In practice, close alignment between the deficit distribution and the operative field should lower the threshold for early specialty consultation.

In most real-world cases, these mechanisms overlap [9,10]. A patient may begin with baseline vulnerability, then experience prolonged positioning with compression and mild traction, compounded by systemic stressors such as hypotension, together producing a clinically evident neuropathy [10]. This “cumulative stress” framing links the mechanism to prevention: reducing total nerve stress across the perioperative course is often more feasible than isolating a single cause. This multifactorial view supports a prevention strategy focused on reducing cumulative “nerve stress” across the entire perioperative course rather than targeting any single factor in isolation [7,9].

Regional Anesthesia-Related Considerations

Regional anesthesia can make early postoperative assessment more challenging and, in a smaller subset of cases, may contribute directly to nerve injury [20]. When sensory symptoms persist beyond the expected duration of the block, new or worsening motor deficits appear, or severe neuropathic pain develops after block resolution, a focused reassessment is warranted [20]. Plausible mechanisms include needle- or catheter-related trauma, inadvertent intraneural injection, and compressive complications such as hematoma, particularly in patients receiving anticoagulation or with underlying coagulopathy [20,21]. Chemical injury from local anesthetic neurotoxicity is less common. Still, it may be considered when symptoms are diffuse within the block distribution, arise without an obvious compressive explanation, and follow higher-concentration exposure or prolonged infusion [20,21]. Importantly, regional anesthesia does not exclude concurrent positioning-related injury; rather, it may delay recognition of harmful pressure or stretch. Diminished sensation during anesthesia can delay recognition of harmful pressure points or sustained stretch, reinforcing the need for careful positioning, proactive padding, and postoperative neurologic surveillance [7,9].

Patient-Related Risk Factors

Host factors influence nerve resilience to perioperative stressors [10]. Diabetes mellitus and other metabolic conditions can produce baseline microvascular compromise and subclinical neuropathy, lowering the threshold for symptomatic injury [28]. Pre-existing entrapment neuropathies (e.g., ulnar neuropathy at the elbow, carpal tunnel syndrome) or prior radiculopathy can predispose patients to postoperative symptom emergence with relatively minor additional insult [16,29]. Low body mass index may reduce protective soft-tissue padding over vulnerable nerve sites, thereby increasing the risk of compression. In contrast, obesity may complicate positioning and increase pressure at certain interfaces [24]. Additional risk factors include thyroid disease, nutritional deficiencies, alcohol-related neuropathy, and advanced age, which can be associated with reduced regenerative capacity and increased comorbidity burden [24].

These factors are clinically relevant because they support individualized risk stratification and informed perioperative counseling [24]. For rehabilitation teams, awareness of baseline risk also frames prognostic expectations and encourages early protective strategies (e.g., orthoses, activity modification) when symptoms develop [16].

Procedure-Related Risk Factors

Procedure-specific factors often shape both the intensity and the duration of “nerve stress” during surgery [9]. In everyday clinical terms, operative time is a useful proxy for risk: the longer the case, the greater the cumulative exposure to compression, traction, and periods of relative hypoperfusion [9,10]. Positioning remains one of the most modifiable contributors [9]. Lithotomy and steep Trendelenburg can increase susceptibility to lower-limb injury through predictable compression points, hip flexion/abduction, and prolonged immobility. In contrast, prone positioning or marked arm abduction may place the upper limb at risk through brachial plexus traction or localized compression [9]. Devices commonly used to maintain position, such as arm boards, shoulder braces, stirrups, and restraints, can become focal pressure sources if padding is inadequate or if positioning is not periodically rechecked during long procedures [9,10].

Tourniquets impose a combined compressive and ischemic burden, with the risk increasing as inflation pressure and duration increase [12,30]. Retractors can also injure nerves adjacent to the operative field by direct compression or sustained stretch, particularly in complex operations where exposure is limited or tissue manipulation is extensive [10,11]. Importantly, the perioperative window does not end at skin closure: postoperative contributors, such as tight dressings, casts, edema, or hematoma, may precipitate or exacerbate neuropathy, underscoring the need for prevention and surveillance well into recovery [9,11].

“Double-Crush” Concept in Perioperative Settings

The double-crush concept suggests that a nerve already under stress at one point, whether from local entrapment or a systemic predisposition, may be poorly tolerated by a second insult elsewhere along its course [29]. In perioperative care, this model can be helpful even when it is not possible to prove causality in an individual patient [29]. For example, someone with a mild, previously stable ulnar neuropathy may become symptomatic after a long case with sustained elbow flexion or pressure over the medial epicondyle [24]. Likewise, patients with cervical radiculopathy or carpal tunnel syndrome may notice a step-up in postoperative symptoms after positioning-related traction or compression [29]. Clinically, this means that patients with known entrapment neuropathies likely have less physiologic reserve; therefore, prevention should be more deliberate, with careful padding, neutral joint alignment, avoidance of prolonged flexion or abduction, and early postoperative screening for new deficits [24].

For rehabilitation clinicians, the double-crush framework is also useful when evaluating symptoms and setting expectations [16]. Presentations do not always map cleanly to a single lesion, and electrodiagnostic studies may show multilevel involvement, for instance, a distal entrapment superimposed on proximal pathology [16]. Keeping this possibility in mind helps avoid attributing all outcomes to a single perioperative event, supports a more comprehensive treatment plan, and enables counseling that aligns with the likely pace and trajectory of recovery [16].

Common injury patterns and clinical presentation

**Figure 1:**
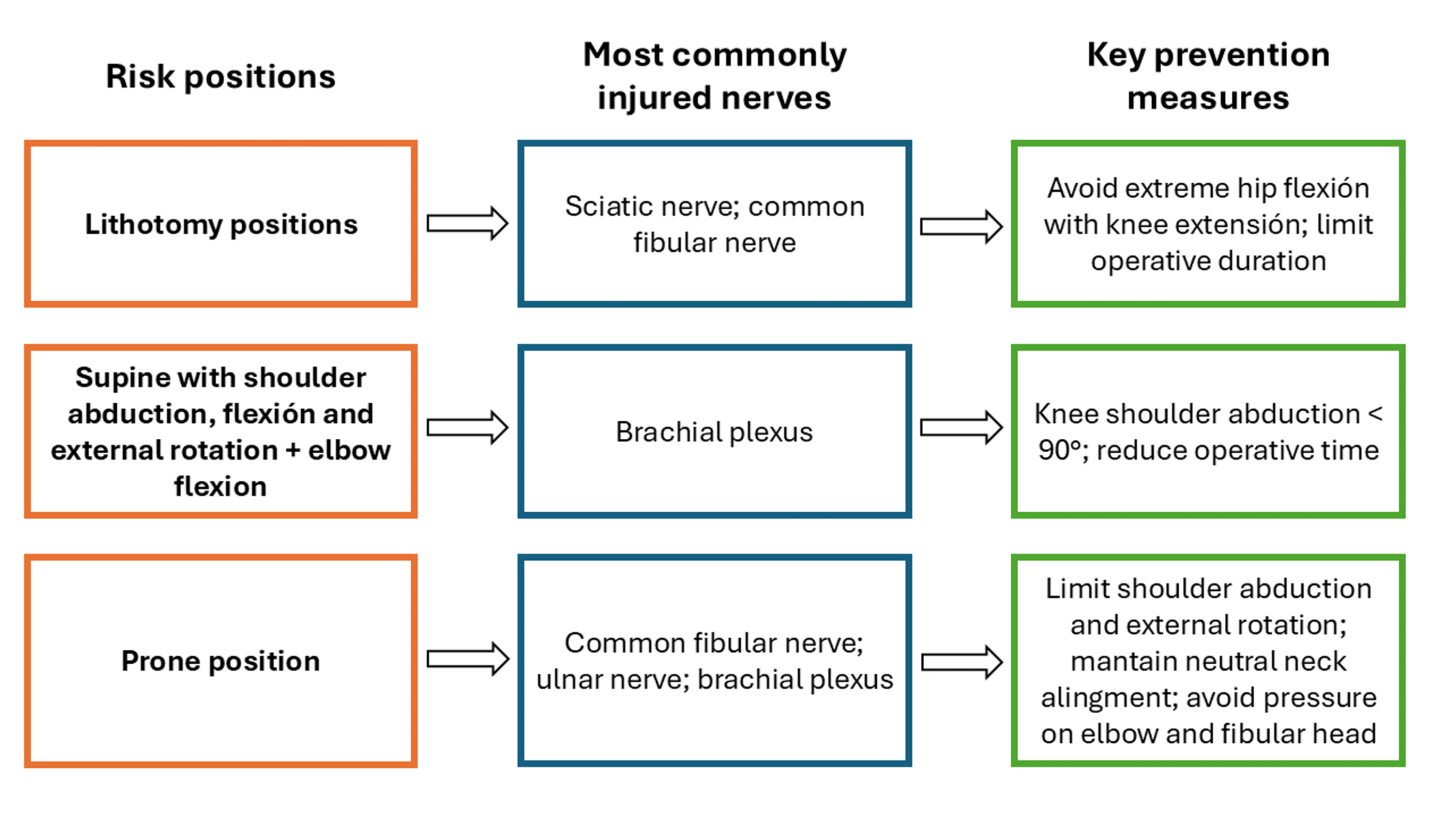
Positioning-related PPNI: risk positions, commonly injured nerves, and key prevention measures PPNI: perioperative peripheral nerve injury

The main nerve injuries of the upper and lower limbs are detailed below. Figure 1 illustrates a schematic representation of positions associated with increased risk of nerve injury, as well as general considerations for their prevention [7,9].

Upper Limb

Brachial plexus injury: Perioperative brachial plexus injury is most often linked to positioning that increases plexus tension, classically, marked shoulder abduction (often beyond 90°), combined with external rotation, posterior displacement of the shoulder, or contralateral neck rotation/extension [7,9]. Prone cases, wide arm placement on arm boards, and insufficient padding can further lower the threshold for injury [7,9]. The clinical picture can range from predominantly sensory complaints to substantial weakness, depending on which trunks or cords are affected [7,9]. Patients may report shoulder or supraclavicular discomfort, paresthesias radiating down the arm, and difficulty with tasks requiring shoulder abduction, elbow flexion/extension, or fine hand function [7,9]. On examination, weakness that spans more than one named peripheral nerve distribution should prompt consideration of a plexopathy rather than an isolated mononeuropathy [7,9,16].

The early differential diagnosis often includes cervical radiculopathy, especially when neck pain and dermatomal sensory changes are present, along with central etiologies when deficits are disproportionate or accompanied by upper motor neuron signs [9,16]. Focal mononeuropathies (ulnar, median, or radial) may also coexist and can complicate localization [9,16]. From a rehabilitation perspective, the initial priorities are practical: protect the limb from ongoing traction, preserve shoulder range of motion within a comfortable range, optimize positioning to minimize further plexus tension, and support function with compensatory strategies while diagnostic clarification is underway [7,9].

Prevention pearls include limiting shoulder abduction, avoiding prolonged maintenance of combined abduction and external rotation, keeping the head and neck in a neutral position, and rechecking arm-board placement and padding after major table adjustments or repositioning during long cases [7,9].

Ulnar neuropathy: The ulnar nerve is among the most commonly affected nerves in perioperative neuropathies [24]. Its primary site of vulnerability is the elbow, where it runs superficially in the ulnar groove and can be compressed or stretched, particularly when the elbow is held in flexion for prolonged periods [24]. Risk increases when the arm is positioned with pressure concentrated over the medial epicondyle, when restraints are tight, or when padding is inadequate [9,24]. Patients with pre-existing ulnar entrapment or broader neuropathic risk factors may have an even smaller margin for additional perioperative stress [9,24].

Clinically, patients typically report numbness or tingling in the ring and small fingers, discomfort along the palmar aspect of the hand, and difficulty with grip or fine motor tasks [24]. On examination, there may be sensory loss in an ulnar distribution and weakness of intrinsic hand muscles-often most evident with finger abduction/adduction and, in more advanced cases, weakness in ulnar-innervated forearm muscles [24]. Because postoperative hand weakness is readily attributed to pain, immobilization, or general deconditioning, it is helpful to deliberately test the intrinsic muscles and compare the findings with those of the contralateral side [24].

The differential diagnosis includes C8-T1 radiculopathy, lower trunk brachial plexopathy, and more diffuse peripheral neuropathy [9]. From a functional standpoint, ulnar neuropathy can be particularly limiting: impaired precision grip, key pinch, and dexterity often result in reduced independence in early self-care [24].

Rehabilitation priorities include protecting the elbow from continued compression, avoiding prolonged flexion, particularly during sleep, considering night splinting in a more neutral elbow position when indicated, and initiating task-oriented hand therapy that focuses on compensatory strategies, intrinsic strengthening as recovery allows, and sensory re-education when persistent paresthesia affects function [24].

Prevention pearls include padding the medial aspect of the elbow, keeping the elbows in a neutral or slightly flexed position when feasible, avoiding resting the elbow on hard surfaces, and ensuring that arm boards and straps do not create focal pressure [7,9].

Median neuropathy (including carpal tunnel exacerbation): Perioperative median neuropathy often shows up as new or clearly worsened symptoms consistent with carpal tunnel syndrome, particularly in patients with pre-existing (sometimes silent) entrapment and in settings where perioperative fluid shifts and edema are common [16]. Wrist-level compression may be triggered by tight dressings, straps used with IV boards, prolonged wrist extension or flexion, or simply postoperative swelling [31]. Proximal median neuropathy is less frequent, but it can occur when the forearm is compressed or when traction forces are applied in a vulnerable position [9,32].

Patients typically report numbness or tingling in the thumb, index, middle, and radial half of the ring finger, often accompanied by nocturnal pain and a noticeable drop in hand function [32]. When weakness is present, it most commonly affects thumb opposition and abduction, which can undermine fine manipulation and reduce grip endurance [32]. In the immediate postoperative period, the timing of symptoms and their correlation with dressing changes or sustained wrist positioning can provide particularly useful clues [16,31].

The differential diagnosis includes C6-C7 radiculopathy and brachial plexus involvement [9]. From a rehabilitation perspective, median neuropathy is clinically meaningful because even subtle sensory disturbances can interfere with precision tasks required for self-care and transfers and complicate the use of mobility aids or assistive devices that rely on stable hand sensation and grip [32].

Rehabilitation priorities include reducing external compression at the wrist, maintaining neutral wrist positioning, considering short-term wrist splinting if symptoms are significant, controlling edema, graded activity modification, and hand therapy emphasizing functional retraining and symptom-guided progression [32].

Prevention pearls include avoiding constrictive wrist dressings, maintaining the wrist in a neutral position when secured to boards, and monitoring for postoperative hand swelling that may exacerbate entrapment [9,31].

Radial neuropathy: Perioperative radial neuropathy is less frequent than ulnar or median involvement, but it can still occur, most often from compression of the nerve along the humeral spiral groove [9,33]. This may happen when the arm rests for prolonged periods against an arm board, positioning device, or a hard table edge [9,33]. Clinically, patients may notice sensory changes over the dorsoradial hand and posterior forearm, and, depending on the lesion level, motor involvement can range from mild extensor weakness to a clear “wrist drop” with impaired finger extension [33,34].

The differential diagnosis includes posterior cord brachial plexopathy and C7 radiculopathy [9]. Functionally, the consequences can be immediate: loss of wrist and finger extension disrupts grasp-release mechanics and may compromise safety during transfers or ambulation, particularly when patients rely on walkers, canes, or other assistive devices [34].

Rehabilitation priorities include early use of a wrist-hand orthosis to maintain a functional hand position, prevention of flexion contractures, task-specific training with adaptive strategies, and progressive strengthening as reinnervation occurs [34].

Prevention pearls include ensuring adequate padding along the posterolateral arm, avoiding sustained focal pressure on the humerus, and reassessing arm position after repositioning [7,9].

Lower Limb

Lateral femoral cutaneous neuropathy (LFCN; meralgia paresthetica): The LFCN is particularly vulnerable where it courses near the anterior superior iliac spine and beneath the inguinal ligament, making it prone to compression in the perioperative setting [35]. Risk tends to increase with prolonged hip flexion, external pressure from straps or positioning devices, and abdominal or pelvic procedures, especially when Trendelenburg positioning or elevated intra-abdominal pressure is involved [36]. Clinically, patients often describe a burning, numb, or tingling sensation over the anterolateral thigh without true weakness. Although motor function is preserved, sensory symptoms can be uncomfortable and disproportionately distressing [36].

The main differentials include L2-L3 radiculopathy and femoral neuropathy; preserved quadriceps strength and an intact patellar reflex favor LFCN involvement [36,37]. For rehabilitation teams, the focus is typically on symptom management and reassurance; mobility is generally maintained, but pain and dysesthesia can impair activity tolerance and sleep and may require targeted strategies to sustain patient engagement in recovery [36,37].

Rehabilitation priorities include patient education; avoidance of hip extension or flexion extremes and external compression; application of principles of neuropathic pain management; and graded activity with pacing [5,36,37].

Prevention pearls include minimizing inguinal pressure from straps and devices, optimizing padding, and limiting prolonged positions that increase tension or compression near the inguinal ligament [5].

Femoral neuropathy: Perioperative femoral neuropathy is most commonly observed after pelvic, hip, or lower abdominal procedures, where the nerve may be exposed to retractor-related pressure, postoperative hematoma, or stretch and compression in the inguinal region [38-41]. Patients typically present with numbness over the anterior thigh accompanied by clear quadriceps weakness, and the patellar reflex is often reduced or absent [16,39]. From a functional standpoint, this combination can be highly limiting early in recovery, making transfers, stair climbing, and safe walking difficult and increasing the risk of falls [38].

The differential diagnosis includes L2-L4 radiculopathy and lumbosacral plexopathy [16,39]. Given the functional consequences of quadriceps weakness, early recognition is essential: it enables timely implementation of knee-stabilization strategies (e.g., bracing, gait aids, and targeted strengthening) to support mobility while neurologic recovery progresses [16,39].

Rehabilitation priorities include early use of a knee-stabilizing orthosis when needed to prevent buckling, targeted strengthening within pain tolerance, gait training with appropriate assistive device selection, and fall-prevention education [16].

Prevention pearls include careful retractor placement with periodic release when feasible, attention to padding of the inguinal region, and vigilance for postoperative iliopsoas or retroperitoneal hematoma in at-risk patients [38,40].

Sciatic neuropathy: The sciatic nerve can be injured in the perioperative setting when it is exposed to sustained stretch or pressure, particularly with prolonged hip flexion, external rotation, or immobilization during lengthy procedures [42,43]. Risk is higher in scenarios such as lithotomy positioning and certain hip-related surgeries [10,42]. Clinically, sciatic neuropathy often presents with a mixed pattern: sensory deficits involving the posterior thigh and leg, along with weakness affecting the hamstrings and/or muscles supplied by the tibial and common fibular divisions [10,42,43]. Because this pattern can closely resemble lumbosacral radiculopathy, careful bedside mapping of sensory changes and reflexes is helpful, although clinical overlap is common [10,42,43].

The functional consequences vary with severity and which division is most affected [43,44]. Predominant involvement of the common fibular division often presents with foot drop and instability during the swing phase. In contrast, significant tibial involvement may lead to plantarflexion weakness, reduced push-off, and impaired gait efficiency [43,44].

Rehabilitation priorities include early use of an ankle-foot orthosis (AFO) for foot drop, protection of insensate skin, gait training emphasizing safety and energy efficiency, and progressive strengthening guided by the recovery trajectory [43,44].

Prevention pearls include minimizing prolonged extreme hip positioning, ensuring adequate padding, and reassessing limb support and rotational alignment throughout long procedures [10,43].

Common fibular (peroneal) neuropathy: The common fibular (peroneal) nerve is especially prone to injury at the fibular head, where it lies superficially and has little soft-tissue protection [45,46]. In the perioperative setting, it can be compressed by leg holders, tight bandaging, sustained knee flexion combined with lateral pressure, or direct contact against rigid parts of the operating table [47,48]. Lithotomy positioning and prolonged immobility are classic scenarios in which this mechanism becomes clinically relevant [10,47,48]. Patients typically present with weakness of ankle dorsiflexion and eversion, resulting in foot drop, along with sensory loss over the dorsum of the foot and lateral shin. Functionally, this often translates into gait instability and an increased risk of tripping, particularly during early mobilization [45,46].

The main differentials include L5 radiculopathy and a more proximal sciatic neuropathy [45,46]. Findings that support a common fibular neuropathy include focal tenderness at the fibular head, a weakness profile that disproportionately affects dorsiflexors and evertors, and relative preservation of inversion (tibialis posterior). However, overlap with proximal lesions is common, and careful clinical correlation remains essential [45,46].

Rehabilitation priorities include prompt prescription of an AFO when foot drop is present, fall-risk mitigation, strengthening of preserved muscle groups, and retraining of gait mechanics [45,46], with sensory loss addressed through skin care education and footwear optimization [45,46].

Prevention pearls include padding the fibular head, avoiding sustained lateral pressure on the knee, ensuring that stirrups and leg supports distribute pressure evenly, and reassessing positioning during prolonged cases [10,45].

Bedside assessment and diagnostic workup

Initial Structured Neurologic Exam (Localization-Oriented)

A structured bedside neurologic examination is the foundation of early assessment when a PPNI is suspected [7,16]. The aim is not only to confirm the presence of a true deficit but also to localize it as precisely as possible, distinguishing mononeuropathy from plexopathy, radiculopathy, or a central process, and to establish a clear baseline for follow-up [5,16]. Because the immediate postoperative period can be a challenging window for examination (pain, sedation, delirium, and residual neuromuscular blockade can all obscure findings), it is often advisable to repeat the assessment once the patient is more alert and analgesia has been optimized [7,16]. This repetition is particularly valuable when early findings are subtle yet functionally consequential.

In practice, a focused minimum examination can be both efficient and high-yield [5,16]. This typically includes (I) motor testing of key muscle groups that align with common perioperative neuropathies (e.g., shoulder abduction; elbow flexion/extension; wrist and finger extension; finger abduction; thumb abduction/opposition; hip flexion; knee extension; and ankle dorsiflexion/eversion/plantarflexion), (II) sensory mapping with light touch and pinprick across peripheral nerve territories, not dermatomes alone, (III) deep tendon reflexes when feasible (biceps, brachioradialis, triceps, patellar, and Achilles), and (IV) simple functional observation, such as transfers, gait, and hand use, which can expose subtle deficits that may be missed on isolated strength testing [7,16]. When the clinical picture suggests compression, additional bedside maneuvers can be helpful: gentle palpation or provocation at common entrapment points (ulnar groove, carpal tunnel region, fibular head) and a quick check of limb alignment and joint posture (e.g., sustained elbow flexion, wrist position, ankle alignment) may support the working mechanism and guide immediate preventive adjustments [5,16].

A structured clinical history is just as important as the neurologic exam [7,16]. At a minimum, it should clarify when symptoms began (immediately in the PACU versus later), how they have evolved (stable, improving, or worsening), whether findings are unilateral or bilateral, and how they relate to the expected resolution of any regional anesthesia [7,16]. Procedural details are often especially revealing: the patient’s position (prone, lithotomy, steep Trendelenburg), the use of restraints and padding, operative duration, arm boards or stirrups, tourniquet pressure and inflation time, retractor placement near vulnerable nerve structures, and postoperative dressings or casts [7,16]. Finally, patient-specific factors, such as diabetes, pre-existing neuropathy, or known entrapment syndromes, help refine pre-test probability and set a realistic framework for counseling about evaluation and recovery [5,16].

Red Flags Requiring Urgent Escalation

Because “postoperative neuropathy” is a broad label that can obscure time-sensitive conditions, it is advisable to include red-flag screening as an explicit step in the initial assessment [5,16]. Rapidly progressive deficits, severe pain out of proportion to examination, tense swelling with pain on passive stretch (suggesting compartment syndrome), new bowel or bladder dysfunction, saddle anesthesia, or features concerning for acute spinal cord or cauda equina involvement should prompt immediate escalation [49,50]. Likewise, sudden focal deficits accompanied by cortical signs (such as aphasia, facial droop, or neglect), altered mental status, or upper motor neuron findings should raise concern for stroke or another central process [51]. In patients who received neuraxial anesthesia or are anticoagulated, acute back pain with evolving neurologic deficits warrants urgent evaluation for a neuraxial hematoma [52].

It is also important to remember that a peripheral nerve injury can coexist with postoperative hematoma, ischemia, or device-related compression [7,16]. For that reason, symptoms that are worsening, especially when pain and swelling are increasing, or when there is a clear temporal link to a dressing, cast, or positioning device, should be approached as potentially reversible until proven otherwise [7,16]. The key point is to prioritize diagnoses in which delay is harmful and to escalate rather than observe when progression is evident [16,49].

When and How to Use Imaging (Ultrasound/CT/MRI) in Selected Cases

Imaging is not necessary in every patient with suspected PPNI. Still, it becomes important when the examination suggests a structural problem that may require intervention, when localization remains unclear, or when deficits are severe or clearly progressive [53,54]. In the immediate postoperative setting, the primary role of imaging is to exclude compressive lesions (e.g., hematoma or seroma), procedure-related structural complications near the operative field, and central or neuraxial causes when red flags are present [53,54].

Ultrasound can be a practical first-line option in selected cases because it is rapid, accessible, and well-suited to superficial processes [55]. It may help identify external compression, detect nearby postoperative hematomas, and, when expertise is available, provide supportive information such as nerve swelling or gross continuity [55]. CT is often more helpful for deep collections (including retroperitoneal hematoma) and for evaluating osseous or hardware-related issues [56]. MRI offers the best soft-tissue detail and is particularly valuable when plexopathy is suspected, when neuraxial complications are a concern, or when there is a strong possibility of structural compression of neural elements [53,57]. That said, feasibility can be limited by availability, patient stability, and postoperative hardware [53,57].

A pragmatic approach is to use imaging when the result is likely to change management, for example, suspected compressive hematoma, severe pain with an evolving neurologic deficit, unexplained plexus-level findings, or concern for a central/neuraxial process [53]. When localization is clear and symptoms are stable, imaging can often be deferred in favor of serial clinical examinations and electrodiagnostic testing performed at the appropriate interval [7,16]. In many cases, the most informative approach is to combine careful bedside localization with selected imaging and time-appropriate electrodiagnosis [16,53].

Electrodiagnostic evaluation

Indications and Clinical Questions Answered by NCS/EMG

Electrodiagnostic testing, including NCS and EMG, is a cornerstone in the evaluation of suspected PPNI because it converts a clinical localization hypothesis into objective physiologic data [16,58]. In perioperative practice, the most helpful uses are to (I) confirm localization (mononeuropathy versus plexopathy versus radiculopathy), (II) define the underlying process (conduction block/neurapraxia versus axonal loss), (III) estimate severity and clarify whether involvement is focal or multifocal, (IV) inform prognosis and the expected pace of recovery, and (V) guide rehabilitation planning, such as orthotic needs, the appropriate intensity of strengthening, monitoring for reinnervation, and counseling about the likely functional trajectory [16,58,59]. For clarity, the sections below distinguish what electrodiagnosis can address early from what becomes reliable only after Wallerian degeneration has progressed.

Electrodiagnosis becomes particularly valuable when the bedside examination is limited by pain, postoperative immobility, or incomplete cooperation, or when the presentation is difficult to interpret due to overlapping possibilities (radiculopathy, pre-existing entrapment neuropathy, generalized polyradiculoneuropathy, or plexus-level injury) [16,58]. It can also be pivotal when symptoms persist beyond the expected resolution of regional anesthesia, when there is substantial weakness early after surgery, or when recovery plateaus and a change in management, such as repeat imaging, specialist referral, or consideration of surgical exploration, is being weighed [16,58]. To avoid redundancy, these indications are referenced again in the clinical pathway rather than restated in detail.

Timing: Hyperacute Limitations and Optimal Windows for Detection and Prognosis

The usefulness of NCS/EMG depends on the timing of the study; testing too early is a common reason clinicians are falsely reassured [60]. In the hyperacute window, roughly the first 7-10 days after injury, studies may appear normal or only minimally abnormal, even when the clinical deficit is clear [60]. This reflects the underlying biology: electrophysiologic signs of axonal loss and denervation develop as Wallerian degeneration progresses distally [61]. Early on, NCS amplitudes may appear relatively preserved, and needle EMG may not yet show fibrillation potentials or positive sharp waves [60,62].

In day-to-day practice, an initial study is most informative approximately two to three weeks after symptom onset, when denervation and axonal injury are more likely to be detectable, and localization is typically more reliable [60,63]. A repeat study at ~4-6 weeks can add prognostic value by showing early reinnervation or, conversely, persistent severe denervation [58,59,63]. Serial testing is particularly helpful when the clinical course is atypical, deficits are substantial, or decisions about surgical referral depend on evidence of axonal continuity and recovery potential [16,58].

These timeframes are useful guides, but they are not absolute [58,63]. When an earlier answer is needed, such as clarifying lesion level in a severe deficit, documenting baseline physiology, or separating a pre-existing neuropathy from a new perioperative injury, an early study can still be appropriate [58]. In those situations, it should be interpreted with the explicit understanding that sensitivity for axonal injury is limited in the hyperacute stage and that repeat testing may be necessary [58,60].

Nerve Injury Classification and Clinical Interpretation (Seddon and Sunderland)

A working grasp of peripheral nerve injury classification can facilitate interpretation of perioperative neuropathies, linking bedside findings, electrodiagnostic results, and the likely pace of recovery [58,64]. The Seddon classification is particularly practical because it uses three familiar categories, neurapraxia, axonotmesis, and neurotmesis, that align well with the core electrodiagnostic question of conduction failure versus axonal disruption [64]. In perioperative care, many positioning- or compression-related injuries fall on the neurapraxia end of the spectrum (often with good potential for recovery) or present as mixed lesions. In contrast, direct trauma or severe compression/ischemia is more likely to produce axonal loss and a longer recovery course [61,64]. This framework supports early counseling and helps structure rehabilitation planning: neurapraxia often allows earlier progression of strengthening and functional use, whereas axonotmesis usually requires longer-term pacing, early orthotic support when needed, and close monitoring for reinnervation [64].

The Sunderland classification builds on the same concepts. Still, it adds detail by grading the degree of internal nerve disruption, from isolated myelin injury to progressive involvement of the endoneurium, perineurium, and epineurium [64]. Clinically, this added granularity is useful because it helps explain why some axonal injuries recover predictably through regeneration. In contrast, others recover incompletely due to internal disorganization, scarring, or loss of guiding architecture [64]. Sunderland grade cannot always be determined with confidence from a single early study, but integrating the clinical course with time-appropriate NCS/EMG findings (and imaging when indicated) can provide a reasonable estimate of severity and prognosis [58,64]. In turn, this can guide decisions about rehabilitation intensity, orthotic needs, and when a lower threshold for specialist referral is appropriate [58,64]. Table 1 summarizes the key features of the Sunderland and Seddon classifications, including the underlying pathophysiology and the expected prognosis for functional recovery at each level of nerve injury.

**Table 1:** Summary of the Seddon and Sunderland classifications of peripheral nerve injury. This table integrates the Seddon and Sunderland classification systems, correlating the extent of structural nerve damage with typical clinical prognosis. While the Seddon classification provides a broad pathophysiologic framework, the Sunderland classification offers greater anatomic detail and prognostic refinement, particularly for axonotmetic injuries

Seddon classification	Sunderland classification	Primary anatomic involvement	Typical prognosis
Neurapraxia	Grade I	Focal conduction block (myelin injury only; axon intact)	Complete recovery
Axonotmesis	Grade II	Axonal disruption with endoneurium intact	Usually complete recovery
Axonotmesis	Grade III	Axonal and endoneurial disruption; perineurium intact	Variable recovery; synkinesis possible
Axonotmesis	Grade IV	Axon, endoneurium, and perineurium disrupted (neuroma-in-continuity)	Poor spontaneous recovery; surgery is usually required
Neurotmesis	Grade V	Complete nerve transection, including epineurium	No spontaneous recovery without surgical repair

Patterns Suggesting Neurapraxia Versus Axonal Loss and Prognostic Implications

One of the main advantages of electrodiagnosis is its ability to distinguish conduction failure from axonal disruption, a distinction that directly informs prognosis and rehabilitation planning [17,58]. Findings consistent with neurapraxia or conduction block, such as focal conduction block or pronounced slowing across a vulnerable segment with relatively preserved distal amplitudes, generally indicate a faster and more favorable recovery once the offending pressure or stretch is relieved [17,58]. In these cases, rehabilitation often emphasizes early functional use, gradual strengthening as tolerated, and symptom-limited activity, while avoiding recurrent compression or traction at the involved site [58,64].

By contrast, axonal loss is suggested by reduced compound muscle action potential amplitudes (and/or sensory nerve action potential abnormalities when applicable), denervation activity on needle EMG in affected muscles, and reduced recruitment with voluntary activation [17,58]. Axonal injury typically implies a longer recovery timeline, with improvement driven by regeneration and reinnervation, often evolving over months rather than weeks [58,61,64]. For rehabilitation teams, this information supports earlier use of compensatory strategies (orthoses, adaptive equipment, and task modifications), proactive prevention of secondary complications (contractures, overuse syndromes, and falls), and the development of strengthening plans that are paced to match the underlying biology of recovery [58,61,64].

Electrodiagnosis can also reveal multilevel or multifocal involvement, which is particularly relevant in patients with metabolic risk factors or pre-existing entrapment neuropathies, essentially a clinical “double-hit” scenario [29,58]. Identifying a coexisting entrapment (such as carpal tunnel syndrome or ulnar neuropathy) in addition to a more proximal lesion helps avoid partial or misdirected management. It supports a more comprehensive rehabilitation plan [29,58].

When to Repeat Studies and How Results Change Rehabilitation Planning

Repeat electrodiagnostic testing is worth considering when the initial study is performed very early, when the clinical trajectory does not match expectations, or when the extent of recovery will materially affect management [16,58,63]. A practical strategy is to repeat testing at ~4-6 weeks if the first study was hyperacute or equivocal and to consider a further reassessment at ~10-12 weeks in more severe injuries, particularly when documenting reinnervation, persistent denervation, or ongoing conduction block could influence specialist referral, surgical decision-making, or long-term rehabilitation planning [16,58,63].

Electrodiagnostic results can also translate into concrete rehabilitation decisions [58,64]. When findings suggest conduction block and the clinical picture is improving, rehabilitation can focus on restoring normal movement patterns, minimizing protective guarding, and progressively loading weakened muscles while maintaining good ergonomics and nerve protection [58,64]. When there is clear evidence of axonal loss, the emphasis often shifts toward early compensatory support, such as an AFO for foot drop or a wrist-hand orthosis for radial palsy, along with energy-conserving gait strategies, strengthening of preserved muscle groups, and patient education about the expected time course of recovery [58,64]. In both scenarios, serial bedside and functional reassessment remain essential: electrodiagnosis provides physiologic clarity but does not replace functional measures, pain evaluation, or participation-based goals [58]. When used appropriately, electrodiagnostic data can help align patient expectations with objective measures of recovery and support coordinated decision-making among surgical, anesthesia, neurology, and rehabilitation teams [16,58,64].

Management and rehabilitation implications

Immediate Measures: Remove Compression, Optimize Positioning, and Provide Early Support

Initial management of a suspected PPNI should begin as soon as the deficit is recognized; there is no need to wait for full diagnostic certainty before taking sensible, low-risk steps [11,16,65]. The first goal is to address potentially reversible contributors, especially external compression [16]. Dressings, splints, casts, sequential compression devices, and tight straps should be inspected and, when safe, loosened, repositioned, or replaced [16,66]. Positioning should then be optimized to reduce traction and avoid repeated pressure at vulnerable sites, for example, minimizing sustained elbow flexion or ensuring there is no focal pressure over the fibular head [9,16]. When weakness is substantial, early protective equipment can reduce secondary injury and improve safety: a wrist-hand orthosis for radial palsy, an AFO for foot drop, or a knee-stabilizing brace for quadriceps weakness can facilitate earlier participation in mobility training [34,66,67].

Because postoperative pain and immobility can quickly lead to stiffness and deconditioning, it is generally advisable to begin range-of-motion exercises early and to activate unaffected muscle groups as tolerated, while adhering to surgical precautions and avoiding maneuvers that aggravate symptoms [68]. Simple bedside measures, such as elevating an edematous limb, maintaining neutral alignment, and using padding strategically, are often low risk yet can meaningfully improve comfort and support function during the early recovery phase [11,16].

Neuropathic Pain Management and Desensitization Principles

Neuropathic pain can be a major source of disability after perioperative nerve injury, and addressing it early is often essential for meaningful participation in rehabilitation [69,70]. In many patients, a multimodal plan works best, optimizing baseline postoperative analgesia while adding strategies specifically aimed at neuropathic pain, alongside non-pharmacologic measures [69,70]. At the bedside, it helps distinguish nociceptive pain from neuropathic features, such as burning, electric shock-like sensations, allodynia, or hyperalgesia [69,70]. Neuropathic pain commonly responds incompletely to opioid-centered regimens and may require targeted medication options selected according to local practice patterns and the patient’s comorbidities [69,70].

For rehabilitation teams, practical interventions can make a real difference: desensitization, graded exposure to touch, and clear pacing strategies often reduce fear-avoidance and improve tolerance to movement and exercise [69,70]. Attention to sleep disruption, anxiety, and catastrophizing is also worthwhile, as these factors can amplify pain and undermine engagement with therapy [71]. Finally, severe pain that is escalating quickly should trigger reassessment for compressive or ischemic complications rather than being attributed to neuropathy alone [69,70].

Early Rehabilitation: Function-First Strategies, ROM Preservation, and Graded Strengthening

Rehabilitation should begin early and proceed concurrently with the diagnostic workup, with goals informed by the likely lesion physiology and the patient’s surgical precautions [16,68]. Early priorities are typically to (I) maintain joint range of motion to reduce contracture risk, (II) protect insensate skin and other vulnerable structures, (III) support safe mobility and self-care through adaptive strategies and assistive devices, and (IV) preserve overall conditioning and aerobic capacity despite a focal neurologic deficit [68].

A practical “function-first” strategy tends to work well: prioritize task-based training transfers, gait, hand use, and activities of daily living with a graded progression, rather than relying solely on isolated impairment-focused exercises [68]. When weakness compromises stability or safety, orthoses and assistive devices should be introduced early and then adjusted as recovery evolves [34,66-68]. For upper-limb neuropathies, hand therapy can focus on dexterity, grip mechanics, and efficient compensatory strategies, while also reducing the risk of secondary overuse injuries [66,68,69]. For lower-limb neuropathies, gait training should incorporate fall-prevention strategies, energy-efficiency training, and, when indicated, environmental modifications to enhance mobility safety during recovery [66-68].

Strengthening is most effective when it is paced to match the biology of recovery. When neurapraxia is likely, resistance can be advanced as voluntary activation returns, with an emphasis on restoring normal movement patterns and minimizing compensatory strain [16,64,68]. When axonal loss is present, early work often centers on strengthening preserved muscles, maintaining neuromuscular control, and preventing learned nonuse, while anticipating a longer timeline for reinnervation [16,68]. Throughout the process, clear education about the expected course and measurable milestones can reduce anxiety and improve adherence [66,68].

Monitoring Recovery, Functional Milestones, and Return-to-Activity Planning

Because recovery and symptom evolution often unfold over weeks to months, structured follow-up is a key part of care [16,68]. Serial bedside examinations, tracking strength, sensation, reflexes, and day-to-day function, provide the most reliable picture of meaningful change [16,68]. Pairing this with simple, standardized functional measures (selected to match the affected limb and the patient’s priorities) can make progress easier to quantify and help calibrate therapy intensity; examples include gait speed, sit-to-stand performance, grip strength, dexterity tasks, and patient-reported function [64,67,68].

When available, electrodiagnostic data can be folded into milestone planning [16,63]. Evidence of conduction block with early clinical improvement generally supports a faster progression of functional loading and skill retraining [16,61,63]. In contrast, axonal loss typically requires a longer time horizon, with greater emphasis on compensatory strategies, orthotic optimization, and more gradual increases in activity as reinnervation emerges [16,61,68]. Decisions about returning to work, sport, or driving should be individualized and grounded in safety considerations, including fall risk, impaired hand control, sensory loss, variability in pain, and the need for protective devices [16,67,68].

Referral Pathways: Neurology, Reripheral Nerve Surgery, and Pain Medicine

Referral decisions are best anchored in a few practical variables: severity, whether symptoms are progressing, the degree of localization confidence, and how recovery is tracking over time [16,65]. Early input from neurology and/or physical medicine and rehabilitation is often helpful when deficits are substantial, the lesion level is uncertain, or symptoms persist beyond what would be expected from transient postoperative effects [11,16]. A pain medicine consultation can also be valuable when neuropathic pain remains severe and function-limiting despite initial measures, particularly if a multimodal medication plan and behavioral or coping supports are needed to keep the patient engaged in rehabilitation [69-72].

Referral for peripheral nerve surgical evaluation should be considered when there is concern for direct nerve trauma, ongoing compression, severe deficits with little or no meaningful improvement, or electrodiagnostic/imaging findings that suggest limited recovery potential [11,65]. Although specific thresholds vary by institution and injury pattern, a pragmatic guiding principle is to avoid “therapeutic inertia” in severe cases: when clinical and electrodiagnostic trajectories indicate poor spontaneous recovery, timely specialist assessment clarifies options, supports shared decision-making, and may improve outcomes for selected patients [16,65].

Practical clinical pathway

Proposed Stepwise Approach to Suspected PPNI

**Figure 2:**
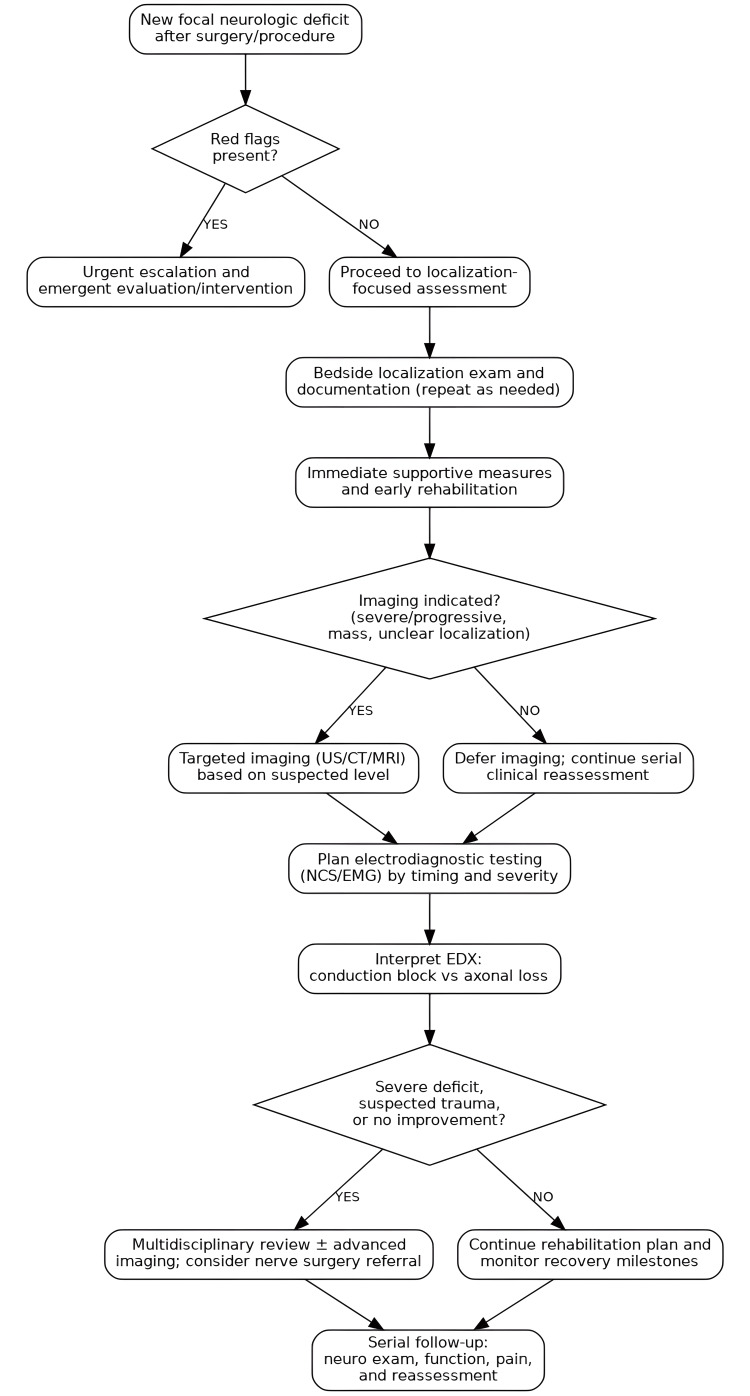
Proposed clinical pathway for suspected PPNI after major surgery, prioritizing urgent triage, bedside localization, early supportive rehabilitation, selective imaging, and time-appropriate electrodiagnostic testing to guide prognosis and management PPNI: perioperative peripheral nerve injury, US: ultrasound, CT: computed tomography, MRI: magnetic resonance imaging, NCS: nerve conduction studies. EMG: electromyography, EDX: electrodiagnostic

A structured clinical pathway can help reduce missed diagnoses and improve coordination across surgery, anesthesia, neurology, and rehabilitation [8]. The approach we propose (Figure 2) rests on three practical principles: (I) quickly ruling out time-sensitive complications, (II) using a localization-focused bedside assessment while providing early functional support, and (III) integrating electrodiagnostic testing at the right time to inform prognosis and rehabilitation planning [8,16]. Individual institutions will adapt the details to local resources, but the overall sequence is broadly applicable across perioperative settings [8,72,73]. This pathway intentionally consolidates key messages from earlier sections to minimize repetition and provide a single, actionable reference.

The pathway begins with a low threshold to take new focal neurologic symptoms seriously [8]. Any new sensory loss, paresthesia, neuropathic pain, or weakness after surgery or an invasive procedure should be treated as clinically meaningful until proven otherwise [7,8]. The first step is triage, looking specifically for features that suggest a central process, neuraxial complications, limb-threatening ischemia, compartment syndrome, or compressive hematoma [8,73]. When these red flags are present, escalation should be immediate, as early intervention may be neurologically decisive [8].

After urgent conditions are excluded, the next step is a localization-oriented neurologic examination with clear documentation to serve as a baseline for follow-up [8]. The history should capture key procedural exposures, positioning, procedure duration, tourniquet use, retractor placement, regional anesthesia technique, and any postoperative devices that could compress superficial nerves [8,73]. Management should proceed in parallel: remove potential external compression, optimize limb positioning, add protective orthoses when indicated (e.g., an AFO for foot drop), and initiate function-focused rehabilitation under surgical precautions [8,16]. In the first postoperative days, serial reassessment is particularly important when pain or sedation limits the reliability of the initial exam [8].

Electrodiagnostic testing is then incorporated based on symptom severity and timing [8,16]. When deficits persist or have a clear functional impact, NCS/EMG is often most informative when performed within a time window that aligns with nerve injury biology, typically around two to three weeks after symptom onset, when questions about axonal involvement and severity can be addressed more reliably [8,16]. Earlier testing may still be appropriate for baseline documentation or when localization uncertainty would change immediate management, with the explicit understanding that sensitivity for axonal loss is limited in the hyperacute phase, and repeat testing may be needed [8,16]. Findings consistent with conduction block generally support a more favorable trajectory and earlier strengthening progression, whereas axonal loss points toward longer-range planning, early orthotic support, and staged rehabilitation guided by reinnervation and functional milestones [8,16].

Finally, the pathway includes clear escalation points [8]. Severe deficits, progressive symptoms, suspected direct trauma, or a lack of meaningful improvement within an expected timeframe should trigger multidisciplinary review, targeted imaging when indicated, and consideration of peripheral nerve surgical consultation [8,16]. Throughout, clear communication with the patient remains essential, aligning expectations with objective findings and reinforcing protective strategies and rehabilitation goals that support recovery [8].

Implementation Notes: Documentation, Prevention Bundle, and Team Communication

Implementing a perioperative nerve injury pathway is easier and more effective when documentation is standardized, and responsibility is shared across teams [73]. At a minimum, clinicians should record the timing of symptom onset, the distribution of sensory and motor findings, reflexes when obtainable, functional limitations, and any external devices that could be contributing to compression [8,73]. Where available, intraoperative positioning checklists and postoperative nursing protocols that include routine limb checks can help prevent avoidable compression injuries [73]. Clear handoffs are particularly important: transitions from the PACU to the ward, from the ward to rehabilitation, and at discharge should include an explicit neurologic summary and practical precautions (e.g., avoiding sustained elbow flexion pressure, protecting the fibular head, using prescribed orthoses, and following fall-prevention measures) [8,73].

A simple “prevention bundle” can be framed in concrete steps: maintain neutral joint positioning, pad superficial nerve sites carefully, reassess positioning during prolonged cases, avoid excessive shoulder abduction and sustained elbow flexion, minimize pressure at the fibular head, and monitor higher-risk patients (such as those with diabetes, known entrapment neuropathies, or prior neuropathy) [7,73]. When a deficit is identified, early interdisciplinary communication helps prevent fragmented care and expedites appropriate referrals, electrodiagnostic testing scheduling, and timely initiation of rehabilitation [8,16].

Limitations of the evidence and future directions

This narrative review has several limitations that reflect both its scope and the broader state of the evidence on PPNIs. First, because the synthesis was targeted rather than systematic, it may not include every relevant study, and the evidence base may be influenced by publication bias and uneven coverage across surgical subspecialties. Second, much of the literature consists of observational studies, case series, and registry or medicolegal reports; these are useful for identifying clinical patterns but limit causal inference and may disproportionately represent more severe presentations. Accordingly, the overall level of evidence in this field remains modest, and current knowledge is derived predominantly from descriptive rather than hypothesis-testing studies. Third, definitions of perioperative nerve injury, reporting thresholds, and follow-up intervals vary widely between studies, making direct comparisons of incidence, risk factors, and outcomes challenging. Fourth, electrodiagnostic timing and interpretation are shaped by real-world factors, pre-existing neuropathy, patient cooperation, postoperative pain, and access to testing, which are not consistently detailed in published reports. Additionally, this review was intentionally focused on peripheral nerve injuries affecting the extremities and did not examine other clinically relevant perioperative neuropathies, such as phrenic nerve injury, in detail. Although beyond the predefined scope of this work, phrenic neuropathy has been reported after cardiothoracic, cervical, and certain regional anesthesia procedures and represents an important area for dedicated investigation.

Future work would benefit from more standardized reporting frameworks and prospective perioperative surveillance protocols that document baseline neurologic status in high-risk patients, capture positioning details in a reproducible way, and include structured postoperative assessments. Pragmatic studies evaluating “prevention bundles” (e.g., positioning checklists, padding protocols, and defined intraoperative reassessment intervals) and their impact on clinically meaningful outcomes are particularly needed. From a rehabilitation perspective, further research should clarify which early interventions, orthotic strategies, targeted therapy approaches, and interventions targeting neuropathic pain pathways most effectively reduce long-term disability and how electrodiagnostic findings can be translated into practical decision tools to inform treatment intensity and referral timing. Finally, multidisciplinary consensus statements that integrate perspectives from anesthesia, surgery, neurology, and rehabilitation could improve consistency in the evaluation and management of patients across institutions.

## Conclusions

PPNIs after major surgery are clinically important because they can leave patients with persistent, function-limiting deficits. These injuries are usually multifactorial and are often under-detected early on, when sedation, pain, immobility, and other postoperative factors can blur the clinical picture and offer competing explanations for symptoms. A structured approach that promptly rules out urgent central or compressive causes, performs a localization-focused bedside assessment, and initiates early protective measures and function-based rehabilitation can improve safety and limit downstream disability.

When symptoms persist or have a clear functional impact, electrodiagnostic testing becomes a valuable extension of the clinical evaluation, provided it is performed in a time window that matches the biology of nerve injury. NCS/EMG can confirm lesion localization, distinguish conduction block from axonal loss, refine prognostic counseling, and guide rehabilitation decisions, including orthotic support and pacing of strengthening and return to activity. Prevention remains a central message: thoughtful positioning and padding, periodic reassessment during long procedures, and heightened vigilance in high-risk patients can meaningfully reduce avoidable “nerve stress.” When injuries occur, early interdisciplinary communication and timely involvement in rehabilitation offer the best chance to maximize recovery and patient-centered outcomes.
